# Fluorescent Probes Based on 7‑(Diethylamino)quinolin-2(1*H*)‑one Cucurbit[7]uril Complexes for Indicator Displacement Assays: *In Silico* and Experimental Approaches

**DOI:** 10.1021/acsomega.5c03501

**Published:** 2025-06-17

**Authors:** Kevin Droguett, Guillermo E. Quintero, Nuno Basilio, Angélica Fierro, Edwin G. Pérez, Margarita E. Aliaga

**Affiliations:** † Departamento de Química Física, Escuela de Química, Facultad de Química y de Farmacia, 28033 Pontificia Universidad Católica de Chile, Santiago 7820436, Chile; § Departamento de Química Orgánica, Escuela de Química, Facultad de Química y de Farmacia, Pontificia Universidad Católica de Chile, Santiago 7820436, Chile; ‡ REQUIMTE/LAQV, Departamento de Química, Faculdade de Ciências e Tecnologia, Universidade Nova de Lisboa, Monte de Caparica 2829-516, Portugal

## Abstract

In this work, two 7-(diethylamino)­quinolin-2­(1*H*)-one derivatives (**DQ1** and **DQ2**) were synthesized and characterized both structurally and physicochemically. The interaction of these derivatives with cucurbit[7]­uril (**CB7**) was explored through combined experimental and *in silico* approaches, highlighting their unique behavior and practical potential. Experimentally, the complexes exhibited a 1:1 stoichiometry, as confirmed by fluorescence spectroscopy and isothermal titration calorimetry (ITC). Remarkably, the interaction with the **CB7** macrocycle resulted in an unusual negative shift in the p*K*_a_ values of the probes alongside a significant enhancement in fluorescence emission, quantum yield, and fluorescence lifetime. From a computational perspective, the molecular dynamics simulations (MD) demonstrated that hydrogen bonds play a critical role in maintaining the system’s stability. Finally, **DQ2** is proposed as a probe for the indicator displacement assay (IDA) and was tested using methyl viologen (**MV**) as an analyte.

## Graphical abstract

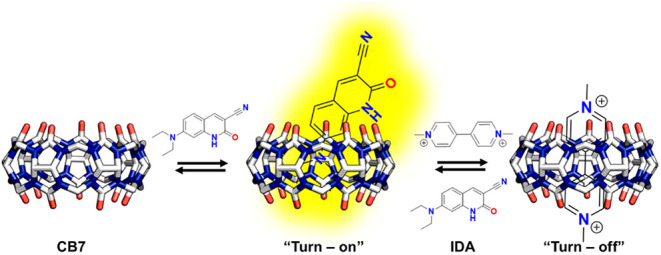

## Introduction

In recent years, supramolecular chemistry has garnered significant attention for various applications.^1–4^ Host–guest inclusion complexes comprise a particular class of supramolecular binding pairs, which have been widely used to construct functional self-assembled architectures. These assemblies involve noncovalent bonding between a host, often a larger structure such as a macrocycle, and a guest molecule that inserts into the host cavity. The inclusion of guest molecules in these confined spaces often results in the modulation of their physicochemical properties relative to those observed in bulk solutions. Commonly employed macrocycles to form host–guest complexes include cyclodextrins,^5^ calixarenes,^6^ pillararenes,^7^ and cucurbiturils.^8^ Notably, cucurbit[7]­uril (**CB7**) has attracted significant interest as a high-affinity host receptor with various applications due to its favorable characteristics, including adequate water solubility, biocompatibility, symmetry, low polarizability, and the presence of a hydrophobic cavity with hydrophilic portals.^9^ These properties make **CB7** a versatile host for numerous applications, including catalysis,^10,11^ drug delivery,^12^ photodynamic therapy,^13^ molecular recognition,^14^ and sensing.^15^

It is noteworthy that the applicability of **CB7** is directly dependent on the specific objectives of the study, which guide the selection of the guest. Consequently, numerous inclusion complexes involving **CB7** have been reported, with guests ranging from metal cations^16^ and small organic molecules, such as aliphatic diamine derivatives,^17^ cyclopentadiene,^10^ and cyclohexane,^18^ to larger molecules like coumarins,^19^ quinoxalinones,^20^ and berberines.^21^ Despite the wide range of structures capable of forming inclusion complexes with **CB7**, quinoline-2­(1*H*)-one derivatives have not been extensively studied from a theoretical or experimental perspective. This family of molecules is aza-analogues of coumarins, with well-documented antibacterial and antifungal properties.^22–26^ Moreover, these derivatives are more thermally and chemically^27^ stable than coumarins, with different optical responses.^28^ Furthermore, incorporating electron-donating group (EDG) and electron-withdrawing group (EWG) into these rings modulates their photophysical properties.^29,30^ In the case of EDG, such as diethylamino (−NEt_2_), and EWG, such as carbaldehyde (−CHO), promote changes in the photophysical behavior of these compounds. An illustrative example is the compound 7-(diethylamino)­quinolin-2­(1*H*)-one-3-carbaldehyde (**DQ1**) (see Figure 1), which contains these functional groups.^31^ A carbaldehyde substituent within the ring facilitates electronic delocalization and enables the modification of the EWG, such as carbonitrile (−CN),^32^ or the addition of other molecular fragments.^30,33^ The presence of this type of substituent on the quinolin-2­(1*H*)-one ring modifies its photophysical properties, which favors the formation of new fluorescent probes. However, their potential^34^ as fluorescent probes, as well as the supramolecular effects induced by **CB7** on properties such as p*K*_a_, absorbance, emission spectra, fluorescence quantum yields, and lifetimes, has not been explored. While several studies have reported the formation of inclusion complexes between **CB7** and coumarin derivatives with binding constants in the range of 10^5^–10^7^ M^–1^,^19,35–38^ it seems reasonable to hypothesize that similar structures, such as quinolin-2­(1*H*)-ones, would also form inclusion complexes with **CB7**. However, no evidence has been presented to confirm such an association.

**Figure 1:**
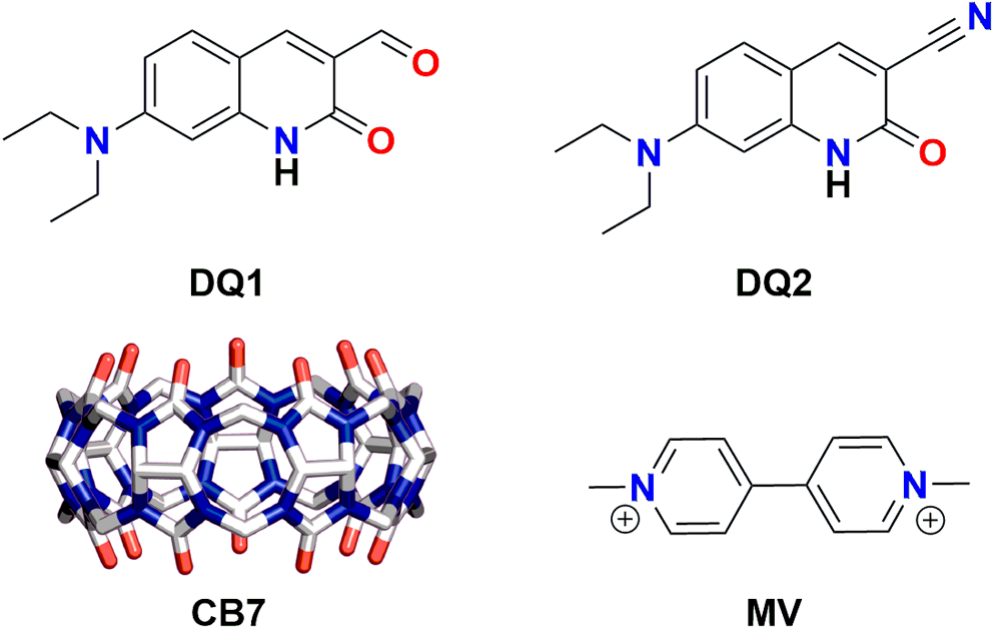
Chemical structures of dyes **DQ1** and **DQ2**, the macrocycle **CB7,** and the competitive guest **MV** employed in this work.

In this work, we investigated the encapsulation of 7-(diethylamino)­quinoline-2­(1*H*)-one-3-carbaldehyde (**DQ1**) and 7-(diethylamino)­quinoline-2­(1*H*)-one-3-carbonitrile (**DQ2**) in the **CB7** cavity (see Figure 1) and the modulation of their properties. Furthermore, we examined the binding thermodynamics to gain insight into the driving force of the complexation. Finally, we studied the use of these quinoline-2­(1*H*)-one derivatives in an indicator displacement assay (IDA) to determine a highly accurate binding constant of 1,1’-dimethyl-4,4’-bipyridinium (**MV**) toward **CB7**.

## Results and Discussion

Physical organic chemistry studies are important for the development of new colorimetric and fluorescent probes for indicator displacement assays (IDA).^39^ To this end, we synthesized and characterized two quinolinone derivatives (Scheme 1, Figures S1–S8 and Tables S1-S4). Moreover, the photophysical characterization of the probes is summarized in Table 1. In particular, Figure 2 shows the absorption spectra of both quinoline-(2*H*)-one derivatives. **DQ1** has a band centered at 430 nm with a molar absorptivity coefficient (ε) of 4.0 × 10^4^ L mol^–1^ cm^–1^, which corresponds to a π→π* transition. Similarly, the π→π* band of **DQ2** is centered at 409 nm. These findings align well with previously reported spectral characteristics of quinolinones and coumarin derivatives, supporting the consistency of their electronic structures and photophysical behavior. The similarity in their absorption bands suggests that both probes possess conjugated systems capable of efficient π→π* transitions, likely influenced by their molecular frameworks and substituent effects

**Scheme 1:**
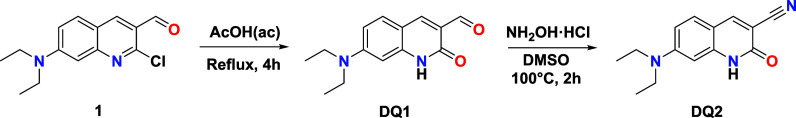
Synthesis of **DQ1** and **DQ2**

**Table 1:** Spectroscopic Data of **DQ1** and **DQ2** and Their Complexes with **CB7**

	$\lambda_{\max}^{\mathrm{abs}}$ (nm)	$\lambda_{\max}^{\mathrm{emi}}$ (nm)	ε (L mol^–1^cm^–1^)	ϕ_f_	τ_1_ (B1)/ns	τ_2_ (B2)/ns
**DQ1**	430	499	40000	0.009	<0.02	–
**DQ1**•**CB7**	434	495	42800	0.049	0.11 (43.97)	0.32 (55.97)
**DQ2**	409	456	47500	0.030	0.13 (27.92)	3.64 (72.08)
**DQ2**•**CB7**	416	458	47800	0.540	1.21 (13.68)	3.56 (86.32)

**Figure 2:**
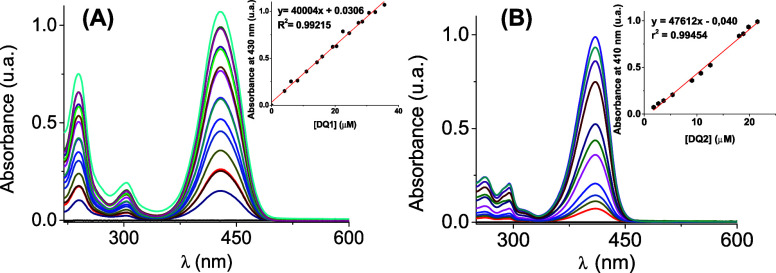
Absorbance spectra of **(A) DQ1** and **(B) DQ2**, in water with 1% of ACN as cosolvent. The inset shows the dependence of concentration with the absorbance at the maximum wavelength for each probe (430 nm and 410 nm, respectively).

In the presence of the macrocycle **CB7**, **DQ1** and **DQ2** exhibited bathochromic shifts in their absorbance spectra of 4 and 7 nm shifts in their emission maxima, respectively. Significant changes were observed in the fluorescence quantum yield and fluorescence lifetimes (see Table 1 and Figures S13 and S14). For **DQ1**, quantum yields increased 5-fold, while **DQ2** demonstrated an exceptional enhancement from 0.03 to 0.54, corresponding to an 18-fold increase in the presence of **CB7**. This improvement in the ϕ_f_ of **DQ2** highlights the profound impact of **CB7** as a host molecule. Such effects have been extensively reported for various molecular systems, where **CB7** enhances ϕ_f_ by stabilizing the intramolecular charge-transfer (ICT) state.^40–42^ This stabilization inhibits the formation of the twisted intramolecular charge-transfer (TICT) state, a nonradiative pathway that typically competes with radiative deactivation. Consequently, **CB7** promotes radiative decay, thereby significantly enhancing the fluorescence efficiency of the guest molecules. These findings underscore the potential of **CB7** in designing highly fluorescent systems for sensing, imaging, and molecular recognition applications.

The fluorescence lifetimes exhibited differences between the two probes. **DQ1** did not exhibit a measurable time decay under the experimental conditions due to a poor fit in the reconvolution analysis; however, it can be inferred that the fluorescence time decay is less than 0.02 ns. In contrast, **DQ2** presented two distinct fluorescence lifetimes: τ_1_ attributed to the locally excited state (LE), and τ_2_ assigned to the intramolecular charge transfer state, with the second lifetime (3.64 ns) contributing most significantly. Notably, **DQ2** in water exhibited high fluorescence lifetimes and ϕ_f_, which can be attributed to the −CN substituent. The nitrile group is a stronger EWG than the −CHO substituent, which improves the photophysical properties of **DQ2** and allows it to be applied as a fluorescent probe.

The complexes formed between the quinoline-2­(1*H*)-one derivatives and macrocycle **CB7** were studied by spectroscopic means. First, the formation of complexes **DQ** *n*•**CB7** leads to an increase in the ϕ_f_ of both probes and stabilizes their second lifetime (see Table 1). This can be attributed to the inhibition of the TICT state of the −NEt_2_ moiety,^43^ thus promoting the planarity of the probes and enhancing their photophysical activity. Furthermore, the above leads to an increase in the fluorescence intensity of the probes upon the addition of the macrocycle, which allowed us to conveniently determine the binding affinity for the complexes, as shown in Figure 3. Both probes have remarkable affinity constants toward **CB7** in the order of 10^7^ M^–1^, which can be useful for their use in indicator displacement assays (IDAs). It is important to note that for those assays, the analyte of interest needs to be in the range given by the equation:^44^

1 $$\log K^{HI}+1\geq \log K^{HG}\geq \log K^{HI}-2$$

where *K* ^HI^ is the probe’s binding constant, in this case, the quinoline-2­(1*H*)-one derivative, and *K* ^HG^ is the constant of the guest of interest. Considering this, the potential analytes for the IDA should be in the range of 10^5^ and 10^8^ M^–1^ binding constants toward **CB7** to perform an adequate binding constant determination.

**Figure 3:**
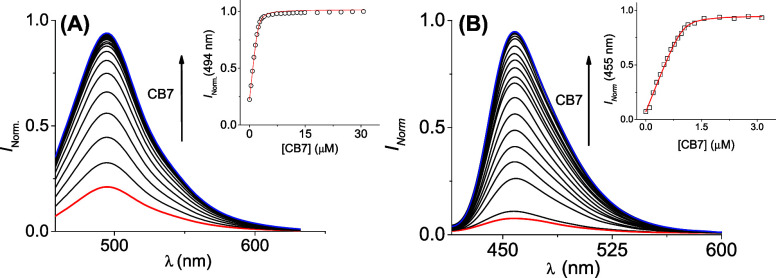
Fluorescent intensity dependence of (A) **DQ1** (1.6 μM) upon the addition of **CB7** (0–70 μM) and (B) **DQ2** (1 μM) upon the addition of **CB7** (0–3 μM). The inset shows the fit for a 1:1 stoichiometry complex (A) (*K*_b_: 7.5 ± 0.8 × 10^6^ M^–1^) and (B) (*K*_b_: 1.1 ± 0.1 × 10^7^ M^–1^). Milli-Q water:ACN (99:1) at 25 °C, pH = 5.86.

After that, the p*K*_a_ values of the quinoline-2­(1*H*)-one derivatives and their host–guest complexes with **CB7** were determined from the absorption spectra recorded at different pH values. As can be observed from Figure 4, both **DQ1** and **DQ2** showed complexation-induced negative p*K*_a_ shifts. It is worth noting that negative shifts are very unusual for **CB7** host–guest complexes, though they have been reported for some compounds with −NEt_2_ substituents.^45,46^ Moreover, the ^1^H NMR spectra (see Figures S9 and S10) presented similar chemical shift displacements for both adducts in the presence of the macrocycle, which suggests a similar binding position inside the cucurbituril. The evidence presented suggests that **CB7** stabilizes the neutral compound over its protonated counterpart when the −NEt_2_ substituent is present.

**Figure 4:**
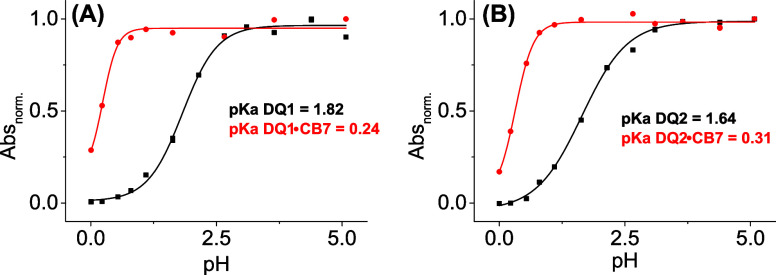
pH titrations of (A) **DQ1** (1 μM) black line and dots and the complex **DQ1**•**CB7** (20 μM of macrocycle) red line and dots and (B) **DQ2** (1 μM) black line and dots and the complex **DQ2**•**CB7** (20 μM of macrocycle) red line and dots in water:ACN (99:1) at 25 °C.

In order to gain further insights into the conformation of the complexes, we conducted molecular dynamics simulations for both systems in explicit water under NTP conditions. The MD results show that both complexes remain stable during the 300 ns simulation (RMSD < 1.5 Å), and a hydrogen bond network contributes to system stabilization (see Figures S3 and S4). Additionally, the conformations of the complexes can be seen in Figure 5, where both complexes show similar behavior. Initially, the quinoline-2­(1*H*)-one derivatives are included by the lactam fraction of the molecules; however, as the simulation progresses, the −NEt_2_ moiety enters the hydrophobic cavity of the macrocycle. Thus, the hydrophilic fraction of the quinolinones is exposed to the solvent, and a hydrogen bond between the N–H of the lactam fraction and the oxygen from the carbonylic portal of the macrocycle is favored. Considering this, the MD simulation suggests that both quinolinones bind similarly to **CB7** in the ground state. Alongside this result is the NMR shift observed in S9 and S10, where an approximate Δδ of 0.7 ppm is noted for the inclusion of each quinolinone in **CB7**. Considering that the quinolinone is partially included, and for a deeper inclusion, like MV inside CB7, a Δδ of 1.5 ppm is observed,^47^ our MD results correlate well with experimental evidence and previously reported −NEt_2_-containing molecules with Δδ values ranging from 0.6 to 0.8 ppm.^35,36,46^ Subsequently, we conducted simulations for the protonated probes; in Figures S19 and S20, an example of the conformation obtained from this study is shown. As observed, both probes are included in the macrocycle via the aromatic side of their core. However, the **DQ2** probe is slightly more included than **DQ1**, favoring the hydrogen bond between the lactam N–H and the upper carbonylic portal, as well as the N–H from −NEt_2_ and the lower cucurbituril rim. A distance measurement between the amino N–H and the portals of the macrocycle during the MD suggests that the **DQ2** probe is more likely to form a hydrogen bond, as shown in Figure 6. In contrast, for **DQ1**, there are fractions of the simulations where this hydrogen bond is not favored, and the −NEt_2_ moiety resides in the middle of the cavity (see Figure 7). Additionally, as illustrated in Figures 6 and 7, the protonated amino group is held inside the cavity of the macrocycle, which suggests that it is not stabilized by the solvent. Thus, the equilibrium is shifted toward the unprotonated quinolinone.

**Figure 5:**
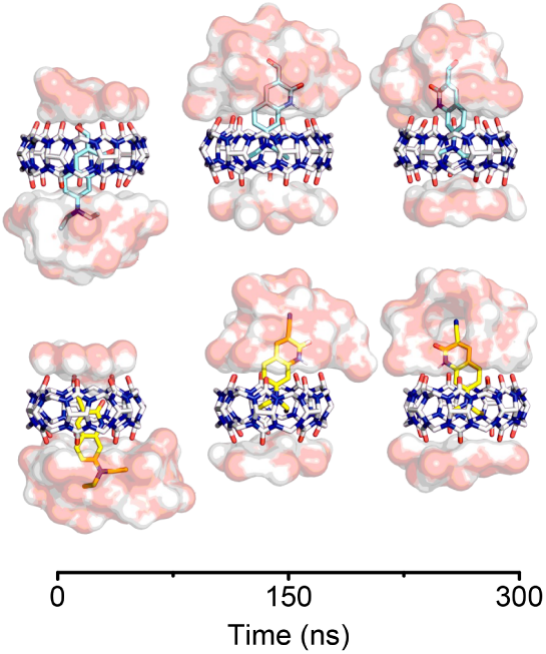
Conformations of (A) **DQ1** and (B) **DQ2** inside **CB7** during a 300 ns MD study. The water molecules interacting with the probes and the macrocycle are shown as a surface.

**Figure 6:**
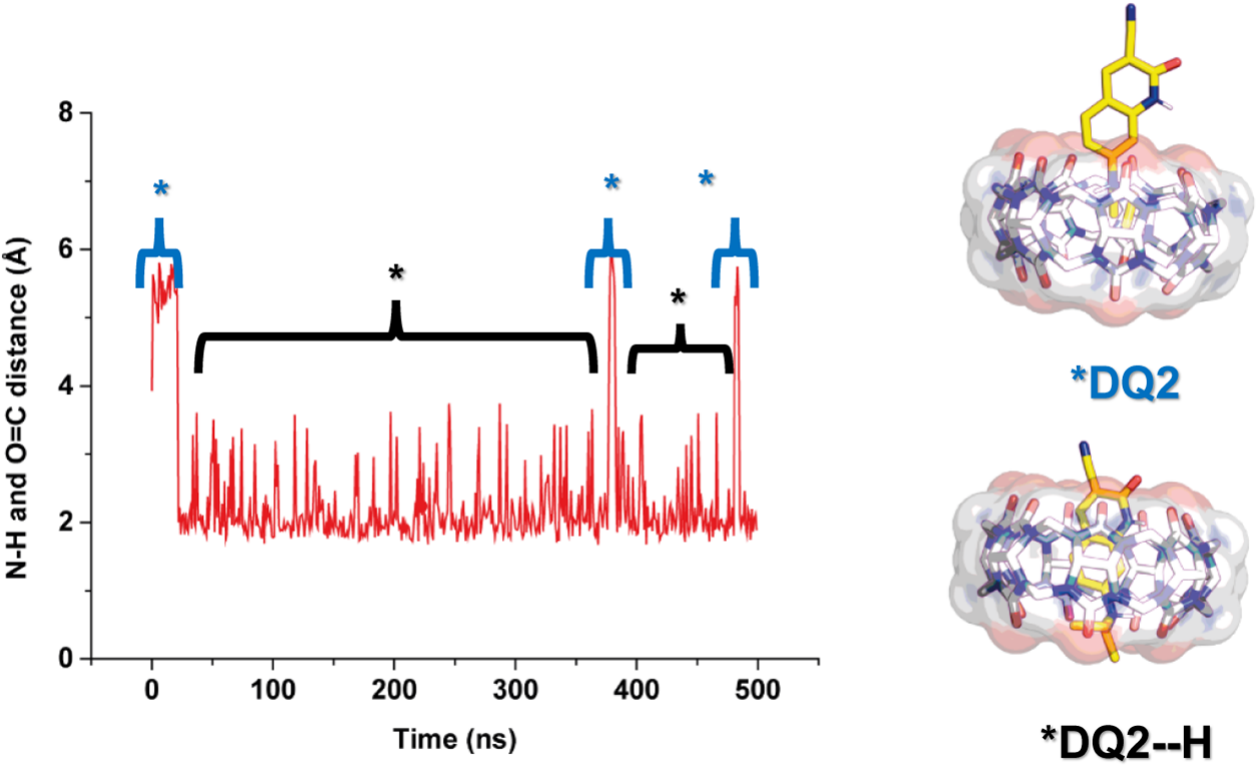
Distance obtained from amino N–H to the portal of **CB7** for protonated **DQ2**. A representative conformation is shown at the right of the graph.

**Figure 7:**
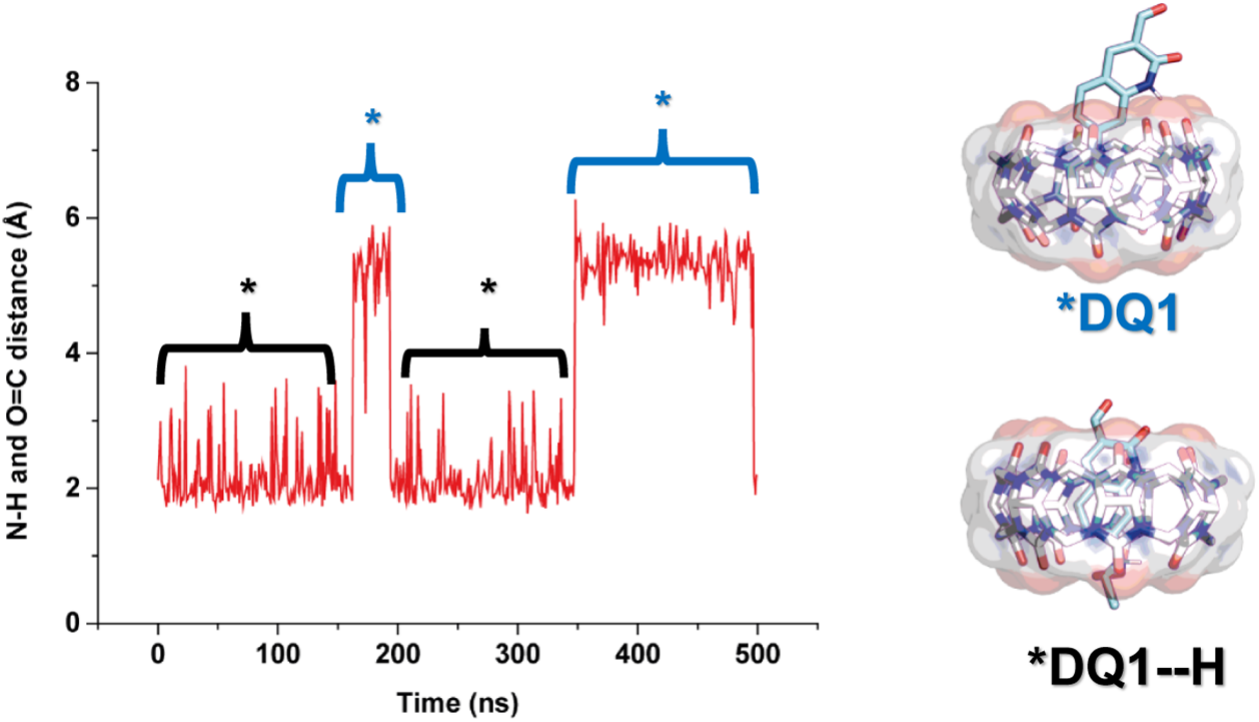
Distance obtained from amino N–H to the portal of **CB7** for protonated **DQ1**. A representative conformation is shown at the right of the graph.

On the other side, the thermodynamic parameters of the complexes were determined using isothermal titration calorimetry (ITC) (see Figure 8), and the results are summarized in Table 2. Due to the poor water solubility of **DQ1** and **DQ2**, we used a 99:1 water:acetonitrile mixture for the macrocycle and the probes. In both cases, the binding is driven by enthalpy, which can be attributed to the release of high-energy water from the macrocycle cavity upon the inclusion of the probe.^48^ On the other hand, the *K*_b_ values from ITC experiments are in good agreement with the constants obtained by fluorescence for both **DQ1** and **DQ2**.

**Figure 8:**
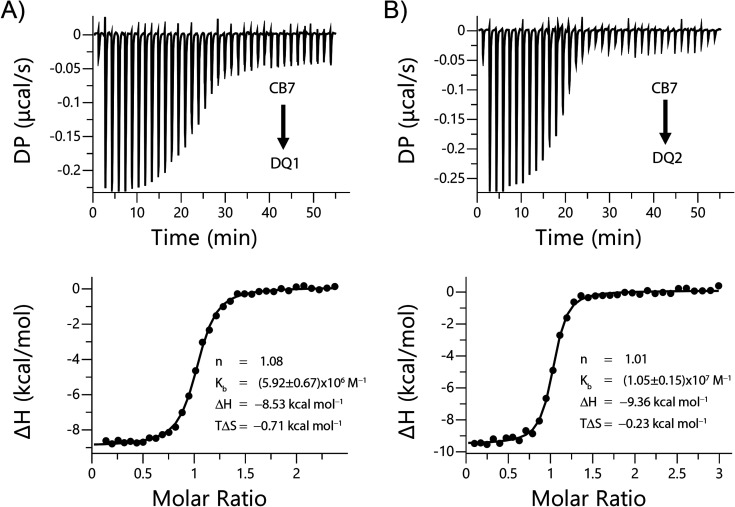
Representative enthalpogram for the binding between **CB7** (100 μM) and (A) **DQ1** (20 μM) and (B) **DQ2** (18.7 μM) in water:ACN (99:1) at 25 °C.

**Table 2:** Thermodynamic Data and Affinity Constant of **DQ1** and **DQ2** with **CB7**

	Δ*H* ^a^ (kcal/mol)	–*T*Δ*S* ^a^ (kcal/mol)	Δ*G* ^a^ (kcal/mol)	Δ*G* ^b^ (kcal/mol)	*K*_b_^a^ (×10^–6^)	*K*_b_^c^ (×10^–6^)	*K*_theo_^b^ (×10^–12^)
**DQ1**•**CB7**	–8.53	–0.71	-9.2 (±0.1)	–17.17	5.9 (±0.7)	7.5 (±0.8)	3.27
**DQ2**•**CB7**	–9.36	–0.23	-9.6 (±0.1)	–18.23	10.5 (±1.6)	11.1 (±0.8)	19.14

a Values obtained from ITC.

b Values obtained from MD.

c Values obtained from fluorescence.

Considering the modulation of the probe’s properties upon the formation of supramolecular complexes, the significant increment in ϕ_f_ of **DQ2** allows us to select it as an indicator for displacement assay. To this end, we study methyl viologen (**MV**), a well-known pesticide, and the supramolecular complex **DQ2**•**CB7**. Upon the addition of **MV**, the fluorescence intensity decreases due to the liberation of **DQ2** and the formation of the **MV**•**CB7** complex. This variation allowed us to determine the binding affinity of **MV** toward **CB7**, as shown in Figure 9 with a value of 1.88 × 10^7^ M^–1^, in contrast with the reported in literature ∼10^5^ M^–1^ in buffer solution^47,49,50^ and 10^6^ M^–1^ in water.^50–52^ Competitive titration (see Figure 10) can be used to quantify the analyte in a range of 0.5 to 3 μM with a limit of detection of 0.24 μM. Considering these promising results, we can propose **DQ2** as a fluorescent probe useful for IDA.

**Figure 9:**
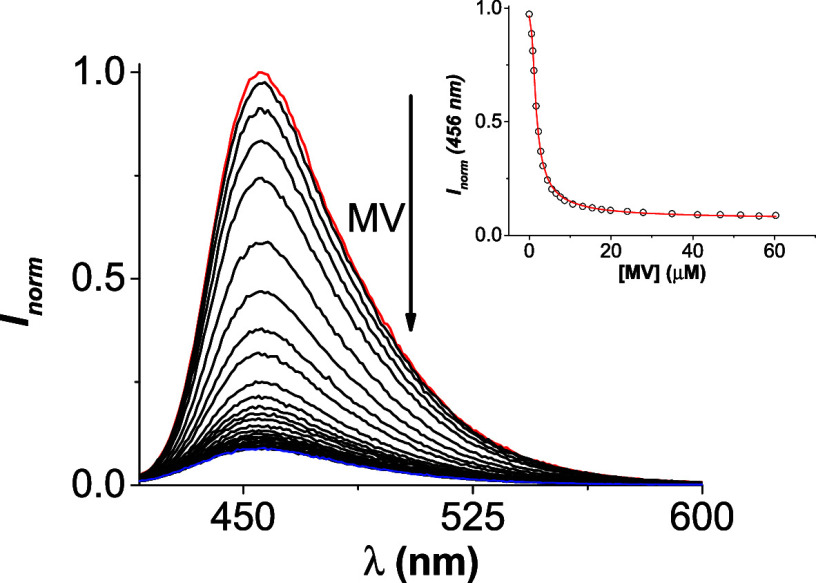
Indicator displacement assay (IDA) using the complex **DQ2**•**CB7** adding methyl viologen (**MV**) as a competitor (*K*_b MV_•_CB7_ = 1.88 × 10^7^ M^–1^) in water:ACN (99:1) at 25 °C.

**Figure 10:**
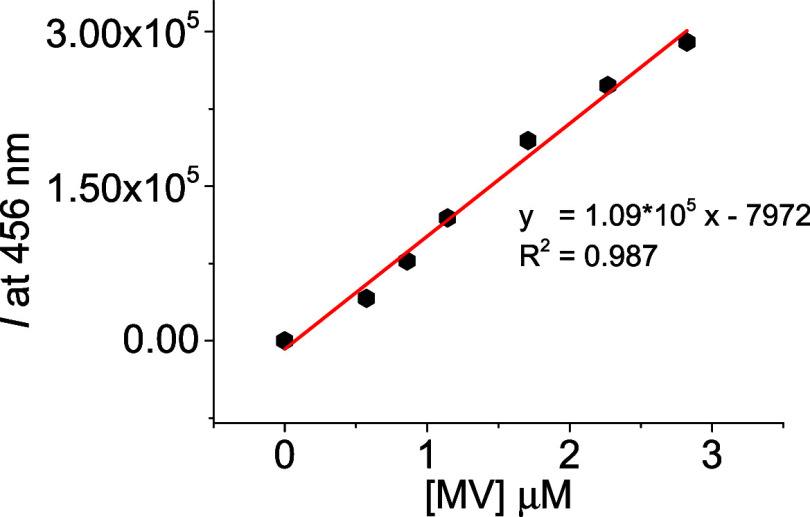
Linear range of the indicator displacement assay (IDA) using complex **DQ2**•**CB7** adding methyl viologen (**MV**) as a competitor in water:ACN (99:1) at 25 °C.

## Conclusions

We synthesized and characterized two quinoline-2­(1*H*)-one derivatives and studied their optical properties and inclusion in the cucurbit[7]­uril cavity. The formation of these supramolecular complexes leads to a modulation in the properties of the quinolinones, increasing the fluorescent quantum yield of the probes and allowing us to determine their affinity toward the macrocycle. This inclusion induces the largest negative Δp*K*_a_ reported to date for 1:1 cucurbituril host–guest complexes. The thermodynamic results showed enthalpy-driven binding due to the liberation of high-energy water and a small entropy gain. Computational results are well correlated with the experimental binding affinity behavior. Finally, the modulation of optical properties and the excellent thermodynamic response toward the inclusion of the probes inside **CB7** allow us to propose the quinoline-2­(1*H*)-one derivative **DQ2** as a new probe for indicator displacement assays.

## Experimental Section

### Materials

Reagents and materials were purchased from Sigma-Aldrich. Curcurbit[7]­uril stock was prepared in ultrapure water (Milli-Q), and the concentration was determined by titration with cobaltocenium cation^53^ by UV–vis spectroscopy.

### Synthesis and Characterization of 7-(Diethylamino)­quinoline-2­(1*H*)-one-3-carbaldehyde (DQ1)

**DQ1** synthesis was carried out according to the methodology reported by Samaan et al.^31^ A mixture of **1** (3.8 mmol, 0.99 g) and 40 mL of 70% aqueous acetic acid was refluxed for 4 h (Scheme 1). The reaction mixture was then allowed to cool to room temperature and poured into ice water. The resulting solid was filtered and washed with several portions of cold water. Finally, the product was purified by column chromatography using dichloromethane:methanol (30:1) as the eluent, yielding a yellow solid (0.65 g, 70% yield). ^1^H NMR (400 MHz, CD_3_CN) δ 9.95 (s, 1H), 8.29 (s, 1H), 7.57 (d, *J* = 9.2 Hz, 1H), 6.80 (d, *J* = 9.3 Hz, 1H), 6.42 (s, 1H), 3.46 (q, *J* = 6.9 Hz, 4H), 1.16 (t, *J* = 2.0 Hz, 6H). ^13^C NMR (101 MHz, CDCl_3_) δ 188.97, 164.92, 152.25, 144.10, 142.01, 132.17, 119.35, 110.39, 110.36, 94.73, 44.96, 12.55. HRMS *m*/*z* [M + H]^+^ calculated for C_14_H_16_N_2_O_2_ 245.1284; found: 245.1290.

### Synthesis and Characterization of 7-(Diethylamino)­quinoline-2­(1*H*)-one-3-carbonitrile (DQ2)

The **DQ2** probe was obtained as described by Chill and Mebane^54^ for the transformation of aldehydes to nitriles. A mixture of **DQ1** (0.4 mmol, 98 mg), hydroxylamine chloride (0.76 mmol, 53 mg), and 2 mL of DMSO was added. The solution is stirred at 100 °C for 2 h, and then, the mixture is cooled to room temperature. The product is extracted with ethyl acetate (3 × 15 mL) and dried with anhydrous sodium sulfate. Then, the solvent was dried under vacuum, and the solid was purified by column chromatography with ethyl acetate as the eluent, yielding 86 mg of a yellow solid (90% yield). ^1^H NMR (400 MHz, CD_3_CN) δ 9.51 (s, 1H), 8.07 (s, 1H), 7.40 (d, *J* = 9.2 Hz, 1H), 6.73 (dd, *J* = 9.1, 2.5 Hz, 1H), 6.40 (d, *J* = 2.5 Hz, 1H), 3.48 (q, *J* = 7.1 Hz, 4H), 1.23 (t, *J* = 7.1 Hz, 6H). ^13^C NMR (101 MHz, CD_3_CN) δ 159.91, 152.38, 147.80, 143.00, 130.49, 117.00–116.00, 109.96, 109.12, 97.65, 94.29, 44.60, 11.71. HRMS *m*/*z* [M + H]^+^ calculated for C_14_H_14_N_3_O 242.1293; found 242.1288.

### Nuclear Magnetic Resonance (NMR) Studies

^1^H and ^13^CNMR spectra were obtained at 25 °C by using a Bruker Avance 400 MHz spectrometer. NMR spectra were processed with MestreNova v14.2 software. Both probes DQ1 and DQ2 were characterized in acetonitrile-*d*_3_, while the complexes DQn•CB7 were prepared by dissolving the probe and the macrocycle in a 1:1 mixture of acetonitrile-*d*_3_ (ACN-*d*_3_) and water-*d*_2_ (D_2_O).

### High-Resolution (HR) QTOF-MS Studies

Mass spectrometry (HR-MS) experiments were conducted using a compact QTOF (Bruker) with an ionization voltage of 6 kV and negative polarity. The scan parameters were as follows: mass range: 50–3000 *m*/*z*, spectra rate: 2 Hz, capillary voltage: 6000 V, nebulizer: 0.6 bar, dry gas: 5 L/min, dry temp: 200 °C.

### Spectroscopic Experiments

UV–vis spectroscopy was conducted using a Cary 60 spectrometer from Agilent Technologies. Similarly, fluorescence spectroscopy was measured using a Horiba FluoroMax-4 fluorescence spectrometer. Both measurements were performed at *T* = 25.0 ± 0.1 °C by using quartz cuvettes with an optical path length of 1 cm and a probe concentration of 1–2 μM.

### Time-Resolved Fluorescence Measurements

Fluorescence lifetimes were measured using a Lifespec II picosecond fluorescence lifetime spectrometer from Edinburgh Instruments. We use a 458 nm laser diode as an excitation source and a fast red-sensitive PMT detector. A total of 10,000 counts were collected to determine the fluorescence lifetime at the fluorescent λ_max_ of each system. The instrument response function (IRF) was recorded by using a diluted Ludox solution to scatter the excitation light. A deconvolution of the IRF alongside the fluorescence decay was performed to obtain the fluorescence lifetimes.^8^

### Determination of Fluorescent Quantum Yield (ϕ_f_)

The fluorescent quantum yield was determined using Coumarin-153 in ethanol as a standard (ϕ_s_ = 0.53).^55^ The dependence of the absorbance and fluorescent emission is recorded for the probes and the standard. Then, using eq 2, quantum yield was obtained:^55^

2 $$\phi_{x}=\phi_{s}\left(\frac{Grad_{x}}{Grad_{s}}\right)\left(\frac{n_{x}^{2}}{n_{s}^{2}}\right)$$

with *x* and *s* being indices for the probe and the standard, respectively, *Grad* is the gradient between the integrated fluorescent emission and the absorbance, η is the refractive index, and ϕ_*s*_ is the quantum yield of the standard. The emission was recorded by exciting at 410 nm with a slit width of 2 nm/2 nm for both the probes and the reference.

### Determination of Binding Affinity Determination (*K*_b_)

The binding affinity was determined using a 1:1 model fitting by fluorescence spectroscopy, according to the equilibrium:

3 H+G⇄HG

*H* corresponds to the host and *G* is the guest, while HG is the complex. Considering that,

4 $$K_{b}=\frac{{[HG]}_{Eq}}{{[H]}_{Eq}\times {[G]}_{Eq}}$$

We can obtain the following quadratic equation for 1:1 model binding:

5 $${[HG]}_{Eq}^{2}-\left({[H]}_{T}+{[G]}_{T}+\frac{1}{Ks}\right){[HG]}_{Eq}+{[H]}_{T}{[G]}_{T}=0$$

where [*HG*]_Eq_ corresponds to the complex in equilibrium and [*H*]_T_ and [*G*]_T_ are the total host and guest, respectively.

6 $$X_{HG}\times I_{HG}+X_{G}\times I_{G}=I_{calc}$$

with *X* being the molar fraction and *I* being the emission intensity of the complex (*HG*) and the guest (*G*). Finally, using eq 6, we can fit the experimental data by minimizing the difference between *I*_calc_ and the experimental emission (*I*_*e*xp_).

### Determination of *K*_b_ by Isothermal Titration Calorimetry (ITC)

Microcalorimetric experiments were carried out using a PEAQ-ITC instrument from Malvern Panalytical. The binding heats between **CB7** and **DQ1** and **DQ2** were studied in water:acetonitrile (99:1) at 25 °C. Data were processed using the PEAQ-ITC Analysis Software, and the integrated heat data were analyzed using ITC data-fitting software.

### Indicator Displacement Assay (IDA)

The competitive assay was carried out using 1,1’-dimethyl-4,4’-bipyridinium (**MV**) as a guest and **DQ2** as an indicator. The binding constant was calculated according to the reactions:

7 $$H+I\overset{K_{1}}{⇄}HI+G\overset{K_{2}}{⇄}HG+I$$

which can be used to derive the following expression using the relevant equilibrium and mass balance equations:

8 $$K_{2}^{2}{[HI]}_{Eq}^{3}+{(2K_{2}+K_{1}-K_{2}K_{1}[H{]}_{T}-3K_{2}K_{1}[I{]}_{T})[HI]}_{Eq}^{2}+\left(K_{2}K_{1}[H{]}_{T}[I{]}_{T}-K_{1}[H{]}_{T}-K_{s1}{[I]}_{T}-K_{2}K_{1}[I{]}_{T}^{2}-1\right)[HI{]}_{Eq}+K_{1}[H{]}_{T}{[I]}_{T}=0$$

where *K*_1_ and *K*_2_ are the binding constants of the indicator and the guest toward the host, respectively.

9 $$\frac{{[HI]}_{Eq}\times I_{HI}+{[I]}_{Eq}\times I_{I}}{{[I]}_{T}}=I_{calc}$$

The resolution of eq 8 allows the determination of [*HI*] (and consequently [*I*]) values, which are required to calculate *I*_calc_ using eq 9. Minimizing the difference between *I*_exp_ and *I*_calc_ allows us to fit the experimental data and optimize the binding constant of **CB7** toward **MV**.

### Computational Methods

The geometry of the molecules was optimized by density functional theory (DFT) at the B3LYP level of theory with the basis set 6-31+G­(d,p) in the software Spartan’14.^56^ The probe•**CB7** complexes were generated by molecular docking using the AutoDock 4.0 suite.^57^ The grid map is centered on the macrocycle with dimensions of 30 × 30 × 30 points and a grid spacing of 0.375 Å. The calculations are based on the Lamarckian genetic algorithm, employing a population size of 150, a maximum of 2.5 × 10 ^6^ energetic evaluations, a maximum of 27000 generations, a mutation rate of 0.02, and an exchange rate of 0.80. The more stable complexes were selected for subsequent molecular dynamics studies.

The former complexes were solvated in a periodic box of explicit TIP3P water. The simulation time was 300 ns for each system. Periodic boundary conditions were applied to the system in the direction of the three coordinates. The simulations were carried out under NTP conditions at 1 atm and 300 K. The software Amber18 ^58^ suite with the AMBER FF14SB force field was used, and the parametrization was done using Antechamber and the LEaP module from AmberTools.^58^

The theoretical binding constants were obtained by a thermodynamic cycle through the molecular mechanical Poisson–Boltzmann surface area (MMPBSA) method of AMBER MD. This methodology considers the solvation of the probe, the macrocycle, and the complex, using the equation:

10 $$\Delta G_{bind,solution}^{{}^{\circ}}=\Delta G_{bind,void}^{{}^{\circ}}+\Delta G_{solv,comp}^{{}^{\circ}}-\left(\Delta G_{solv,probe}^{{}^{\circ}}+\Delta G_{solv,macro}^{{}^{\circ}}\right)$$

with ΔG_solv, comp_, ΔG_solv, probe_, and ΔG_solv, macro_ being the Gibbs free energy of solvation for the complex, the probe, and the macrocycle, respectively, while ΔG_bind, void_ and ΔG_bind, solution_ are the binding free energy for the probe-macrocycle complex in void and solution, respectively.^59^ It is important to mention that enthalpy and entropy were considered in these methods; however, as is known, the entropy value is usually overestimated. Thus, our calculation explicitly approximated the entropy.^60,61^

## Supplementary Material



## Notes

Additional information on characterization and computational studies is available in Supporting Information in Figures S1–S20 and Table S1–S4 (PDF)
