# Supplementary material for: Fluorescent Probes Based on 7‑(Diethylamino)quinolin-2(1H)‑one Cucurbit[7]uril Complexes for Indicator Displacement Assays: In Silico and Experimental Approaches
Source: ACS Omega. 2025 Jun 17;10(25):27550–8. doi: 10.1021/acsomega.5c03501 (PMC12224112; doi:10.1021/acsomega.5c03501)
Supplement: Supplementary file 1 [file ao5c03501_si_001.pdf]

Supporting information for

Fluorescent Probes Based on 7-(diethylamino)quinolin-2(*1H*)-one Cucurbit[7]uril Complexes for Indicator Displacement Assays. In Silico and Experimental Approaches

Kevin Droguett,[a] Guillermo E. Quintero,[a] Nuno Basilio[c], Angélica Fierro,[b] Edwin G. Pérez,[b] and Margarita E. Aliaga.[a]*

[a] Departamento de Química Física, Escuela de Química, Facultad de Química y de Farmacia, Pontificia Universidad Católica de Chile, Chile

[b] Departamento de Química Orgánica, Escuela de Química, Facultad de Química y de Farmacia, Pontificia Universidad Católica de Chile, Chile

[c] REQUIMTE/LAQV, Departamento de Química, Faculdade de Ciências e Tecnologia, Universidade Nova de Lisboa, Monte de Caparica 2829-516, Portugal

Corresponding author: Margarita E. Aliaga; e-mail: mealiaga@uc.cl

Table of Contents

Figure Index

Figure S1. NMR-^{1}H spectra of **DQ1** in acetonitrile-d3. 3
Figure S2. NMR-^{13}C spectra of **DQ1** in chloroform-d....................4
Figure S3. NMR DEPT-135 spectra of **DQ1** in chloroform-d. 6
Figure S4. Positive-mode HRMS spectra of **DQ1**....................7
Figure S5. NMR-^{1}H spectra of **DQ2** in acetonitrile-d3. 8
Figure S6. NMR-^{13}C spectra of **DQ2** in acetonitrile-d3. 9
Figure S7. NMR DEPT-135 spectra of **DQ2** in acetonitrile-d3....................11
Figure S8. Positive-mode HRMS spectra of **DQ2**....................12
Figure S9. NMR-^{1}H spectra of **DQ1** (red) and **DQ1•CB7** (cyan) in a mixture acetonitrile-d3:deuterium oxide 1:1. 13
Figure S10. NMR-^{1}H spectrum of **DQ2** (red) and **DQ2•CB7** (cyan) in a mixture acetonitrile-d3:deuterium oxide 1:1. 14
Figure S11. Variation of **DQ1** absorbance upon the addition of an excess of **CB7**....................15
Figure S12. Variation of **DQ2** absorbance upon the addition of an excess of **CB7**....................15
Figure S13. Dependence of integrated fluorescence intensity and absorbance for coumarin-153, **DQ1** and **DQ1•CB7**, used for the determination of quantum yield of the probe and complex. 16
Figure S14. Dependence of integrated fluorescence intensity and absorbance for coumarin-153, **DQ2** and **DQ2•CB7**, used for the determination of quantum yield of the probe and complex. 17
Figure S15. UV-Vis Spectra of A) **DQ1** and B) **DQ1•CB7** at different pH....................17
Figure S16. UV-Vis Spectra of A) **DQ2** and B) **DQ2•CB7** at different pH....................17
Figure S17. RMSD for **DQ1** and **DQ2** inside **CB7** during a 300 ns MD study. 18
Figure S18. Hydrogen bonds formed between probe - **CB7** (red bars) and probe or **CB7** – solvent (black bars) A) **DQ1**, B) **DQ2** during a 300 ns MD study....................18
Figure S19. Conformations of **DQ1** inside **CB7** during a 300 ns MD study. The water molecules interacting with the probe and the macrocycle are shown as a surface. 19
Figure S20. Conformations of **DQ1** inside **CB7** during a 300 ns MD study. The water molecules interacting with the probe and the macrocycle are shown as a surface. 20

Table Index

Table S1. ^{1}H NMR signal assignment of **DQ1**....................3
Table S2. ^{13}C NMR signal assignment of **DQ1**. 5
Table S3. ^{1}H NMR signal assignment of **DQ2**....................8
Table S4. ^{13}C NMR signal assignment of **DQ2**. 10

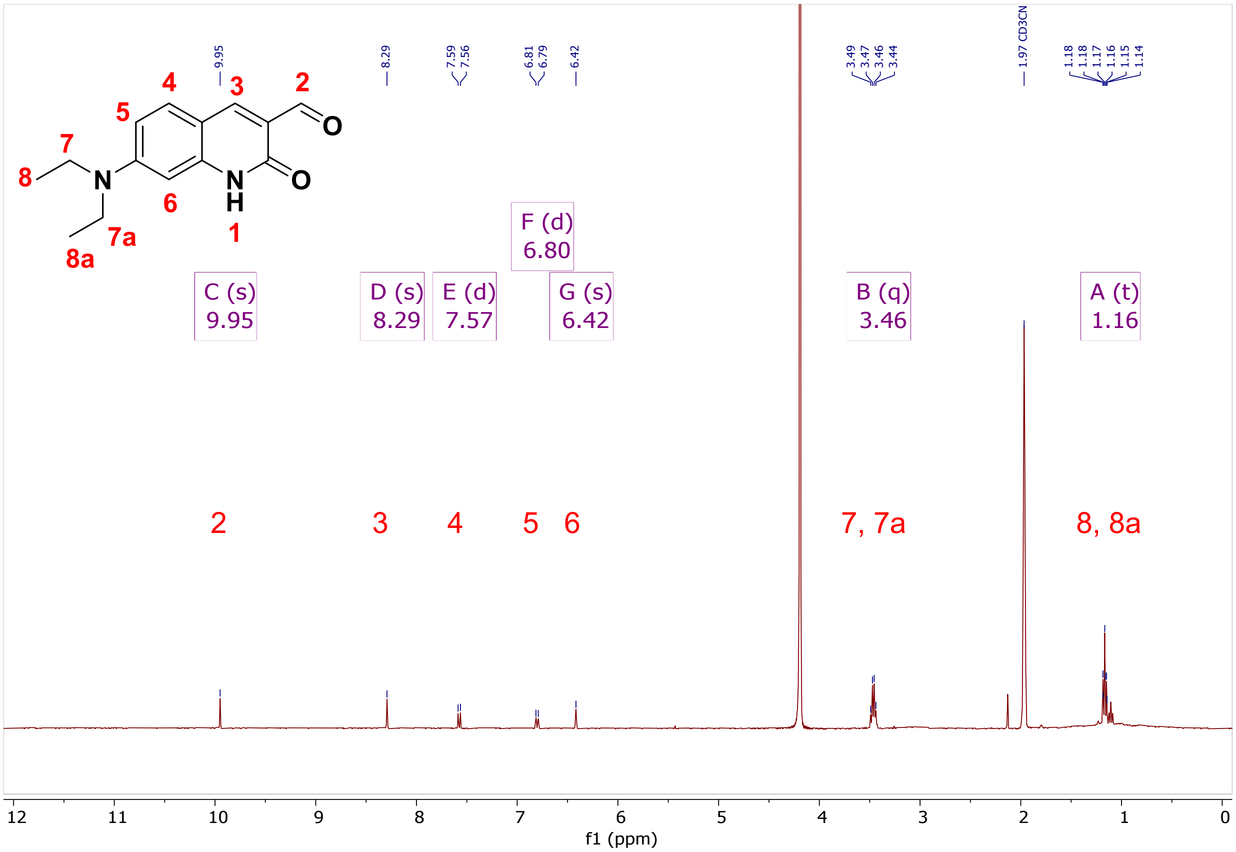

Figure S1. NMR-^{1}H spectra of **DQ1** in acetonitrile-d3.

Table S1. ^{1}H NMR signal assignment of **DQ1**.

Protons	Multiplicity	δ (ppm)
H_2	*s*	9.95
H_3	*s*	8.29
H_4	*d*	7.57
H_5	*d*	6.80
H_6	*s*	6.42
H_7, H_{7a}	*q*	3.46
H_8, H_{8a}	*t*	1.16

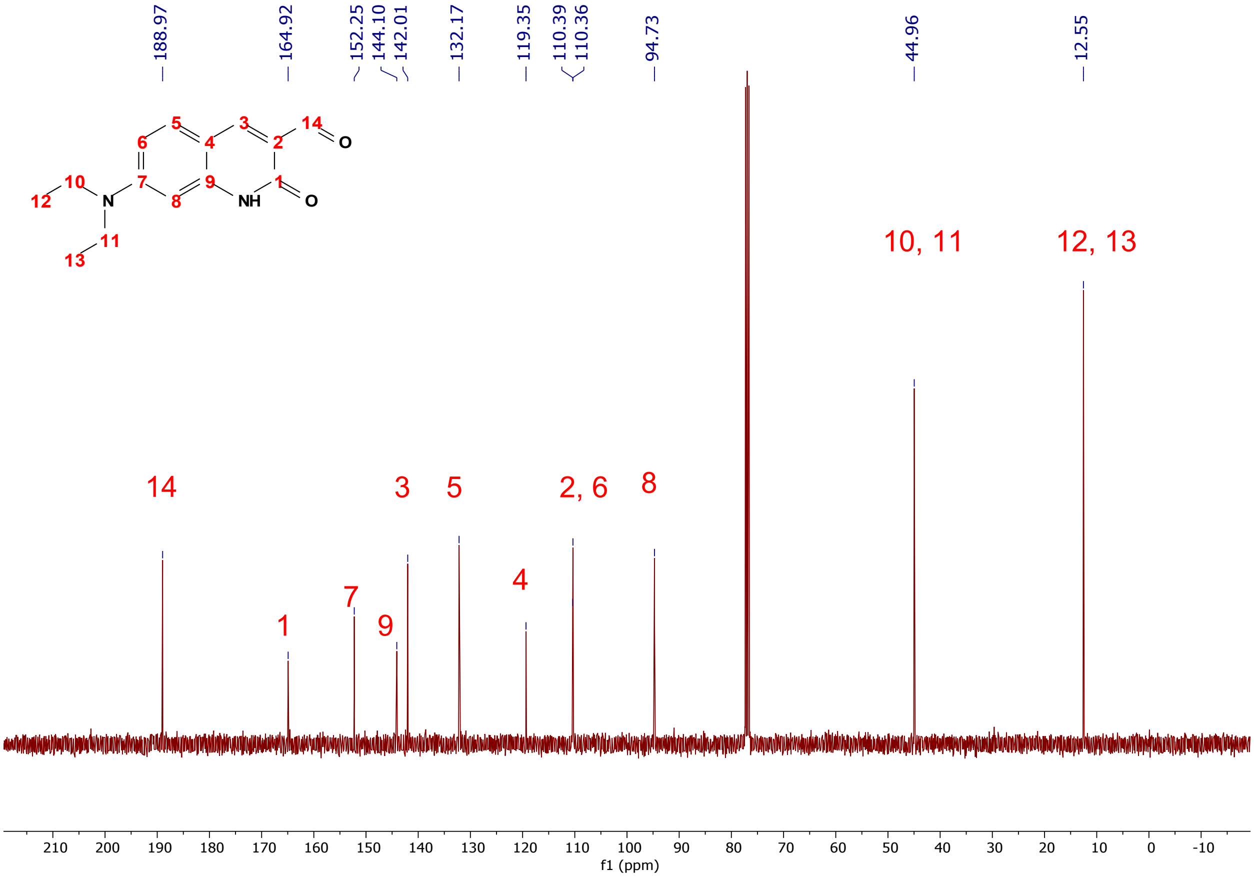

Figure S2. NMR-^{13}C spectra of **DQ1** in chloroform-d.

Table S2. ^{13}C NMR signal assignment of **DQ1**.

Carbons	δ (ppm)
C_{14}	188.97
C_1	164.92
C_7	152.25
C_9	144.10
C_3	142.01
C_5	132.17
C_4	119.35
C_2	110.39
C_6	110.36
C_8	94.73
C_{10}, C_{11}	44.96
C_{12}, C_{13}	12.55

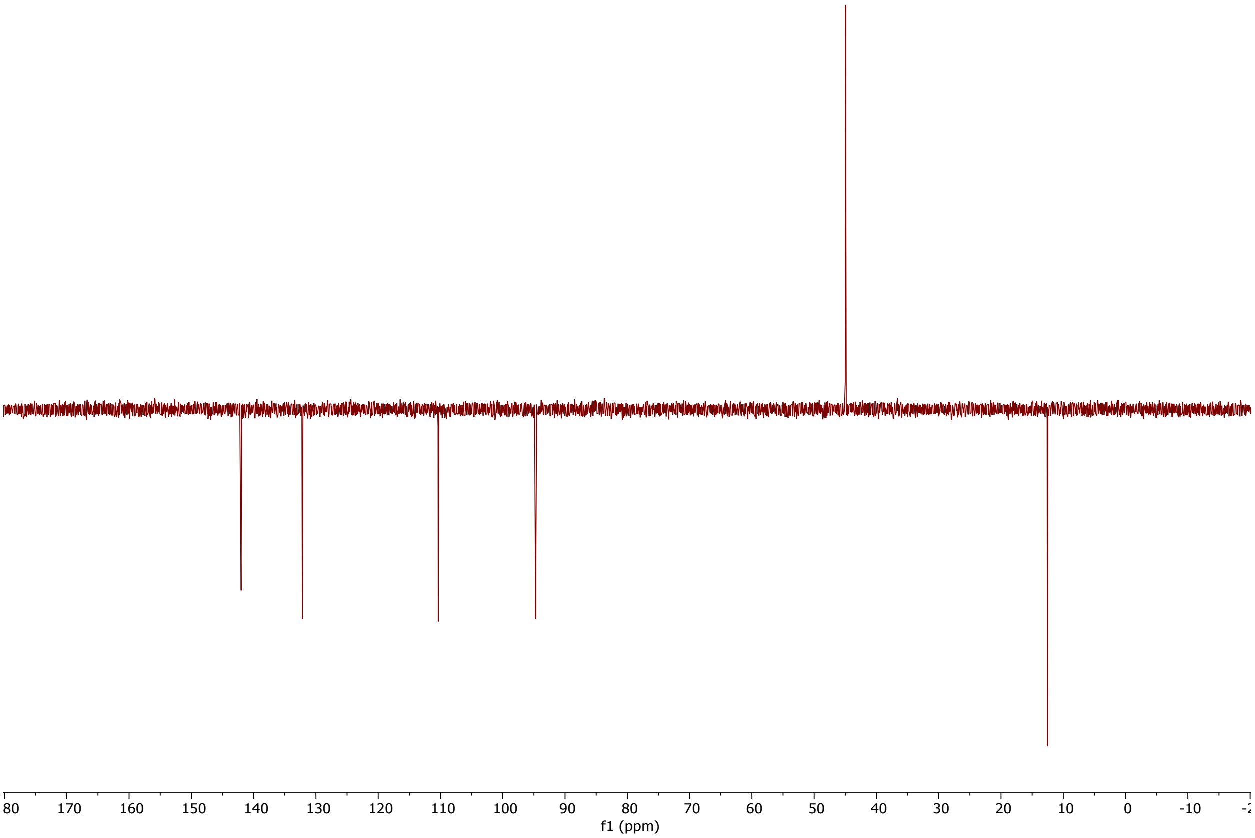

Figure S3. NMR DEPT-135 spectra of **DQ1** in chloroform-d.

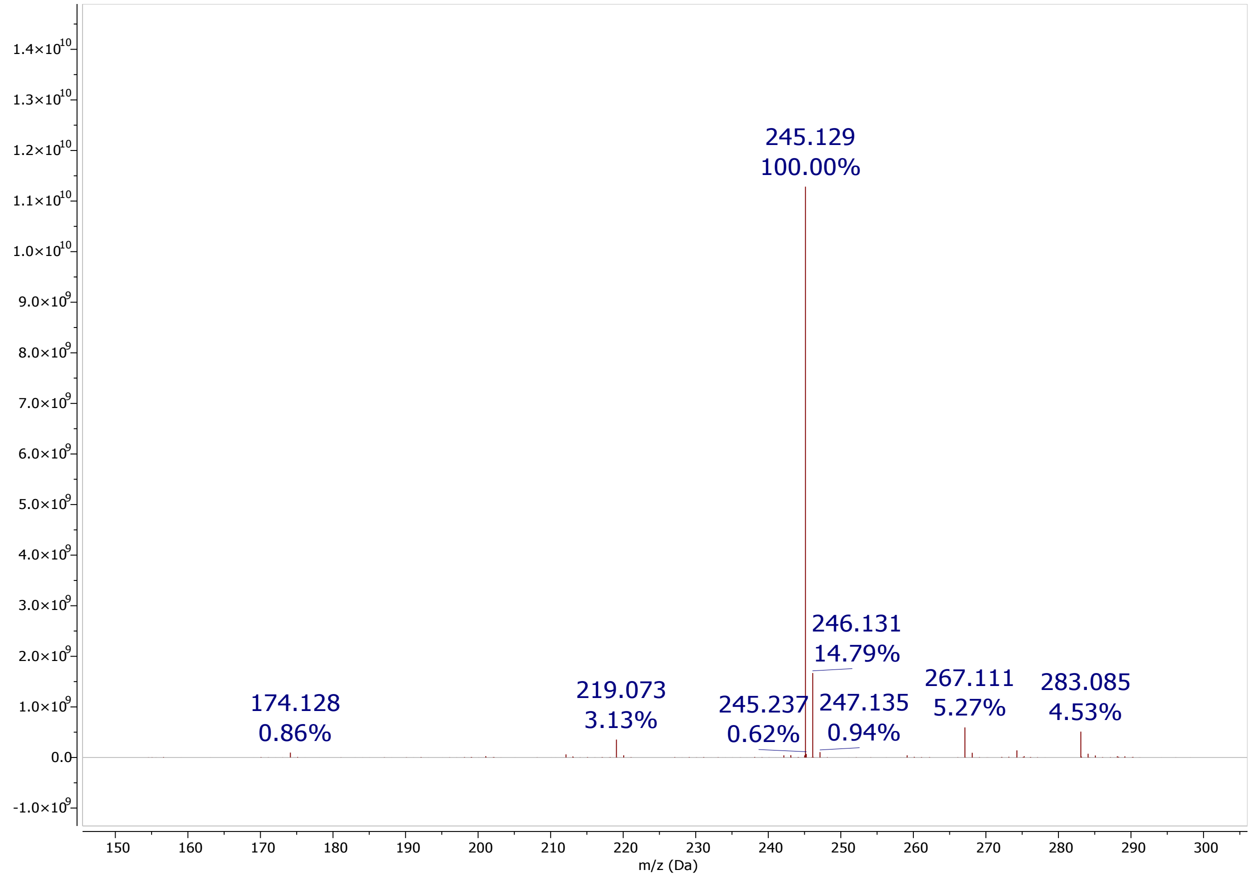

Figure S4. Positive-mode HRMS spectra of **DQ1**.

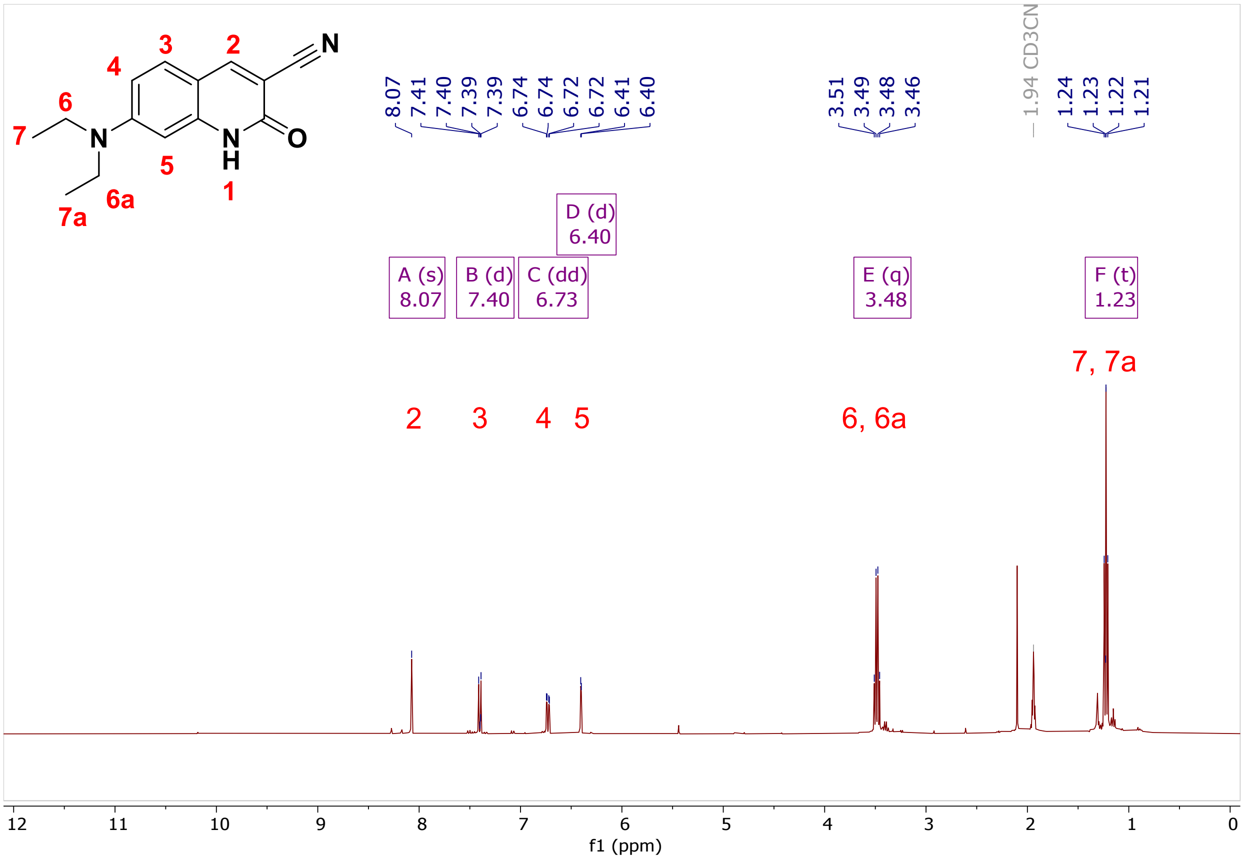

Figure S5. NMR-^{1}H spectra of **DQ2** in acetonitrile-d3.

Table S3. ^{1}H NMR signal assignment of **DQ2**.

Protons	Multiplicity	δ (ppm)
H_2	*s*	8.07
H_3	*d*	7.40
H_4	*dd*	6.73
H_5	*d*	6.40
H_6, H_{6a}	*q*	3.48
H_7, H_{7a}	*t*	1.23

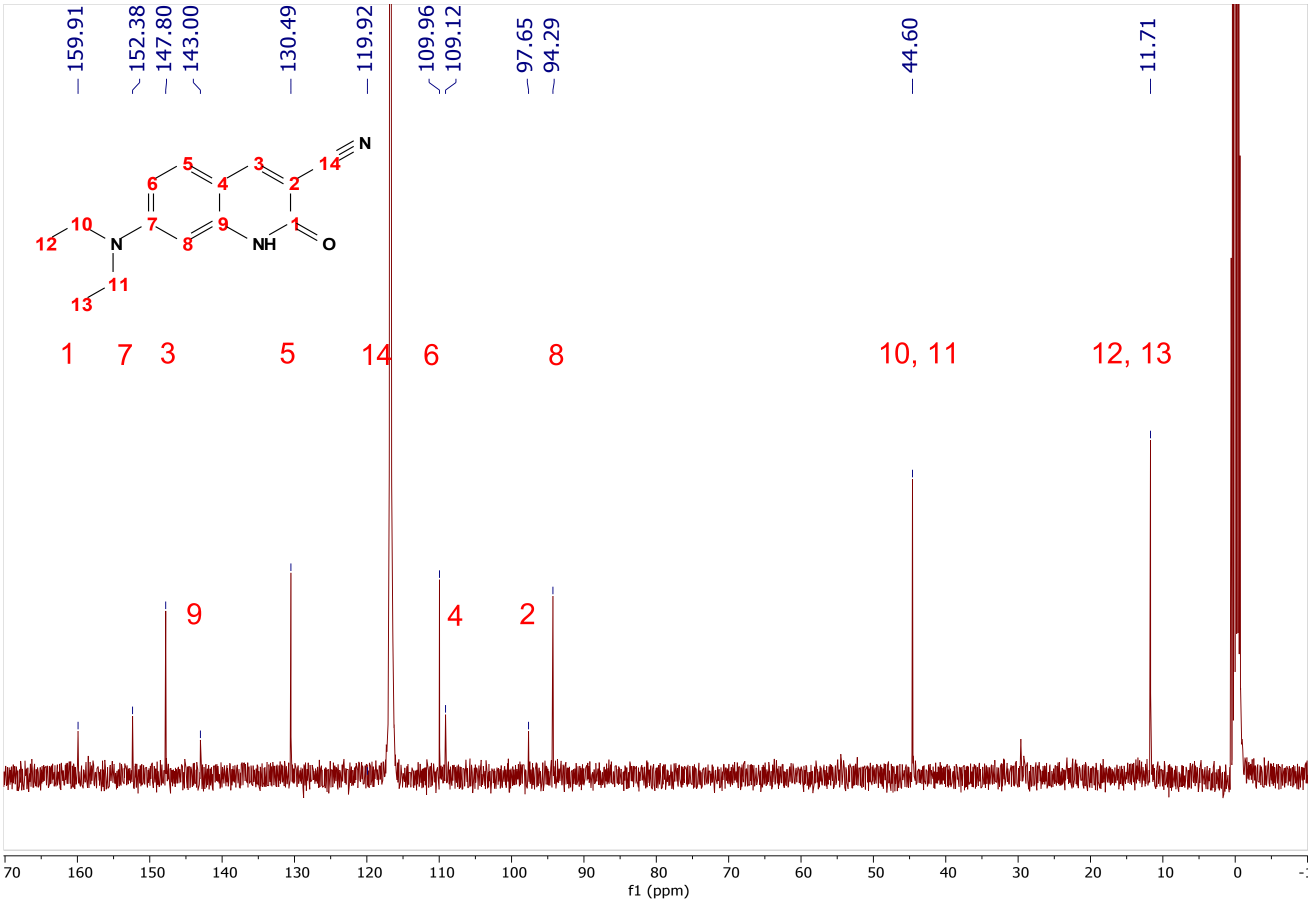

Figure S6. NMR-^{13}C spectra of **DQ2** in acetonitrile-d3.

Table S4. ^{13}C NMR signal assignment of **DQ2**.

Carbons	δ (ppm)
C_1	159.91
C_7	152.38
C_3	147.80
C_9	143.00
C_5	130.49
C_{14}[a]	117.00 – 116.00
C_6	109.96
C_4	109.12
C_2	97.65
C_8	94.29
C_{10}, C_{11}	44.60
C_{12}, C_{13}	11.71

[a]Carbon signal overlapped with the deuterated solvent signal

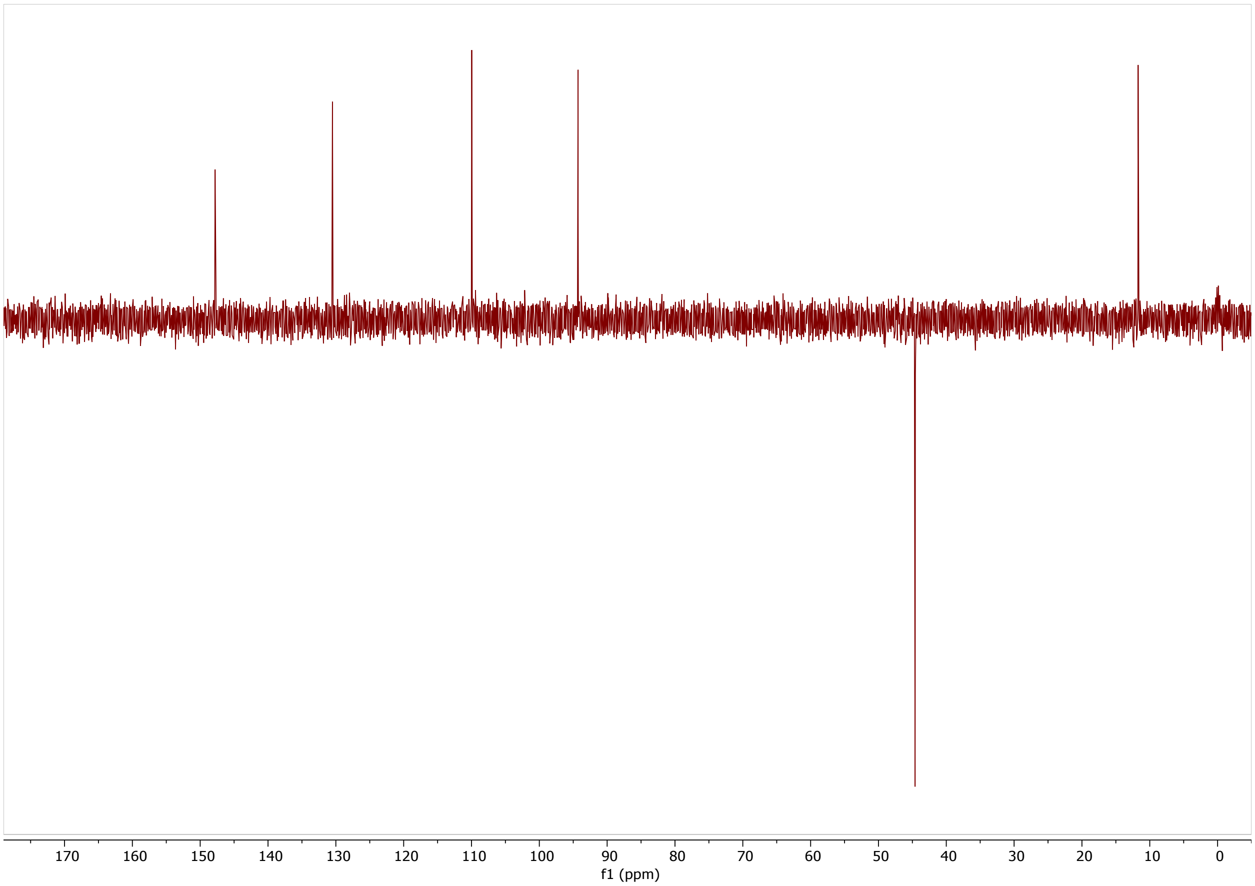

Figure S7. NMR DEPT-135 spectra of **DQ2** in acetonitrile-d3.

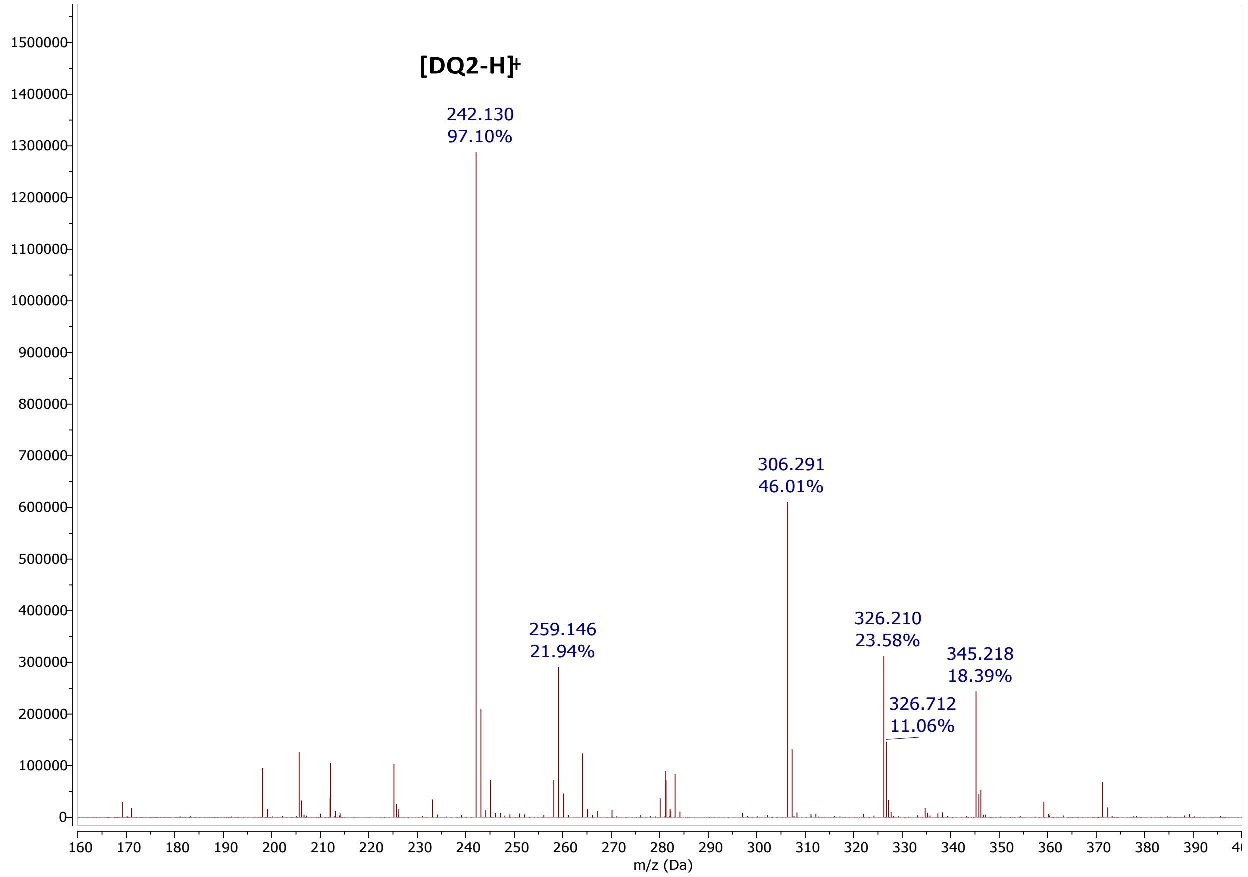

Figure S8. Positive-mode HRMS spectra of **DQ2**.

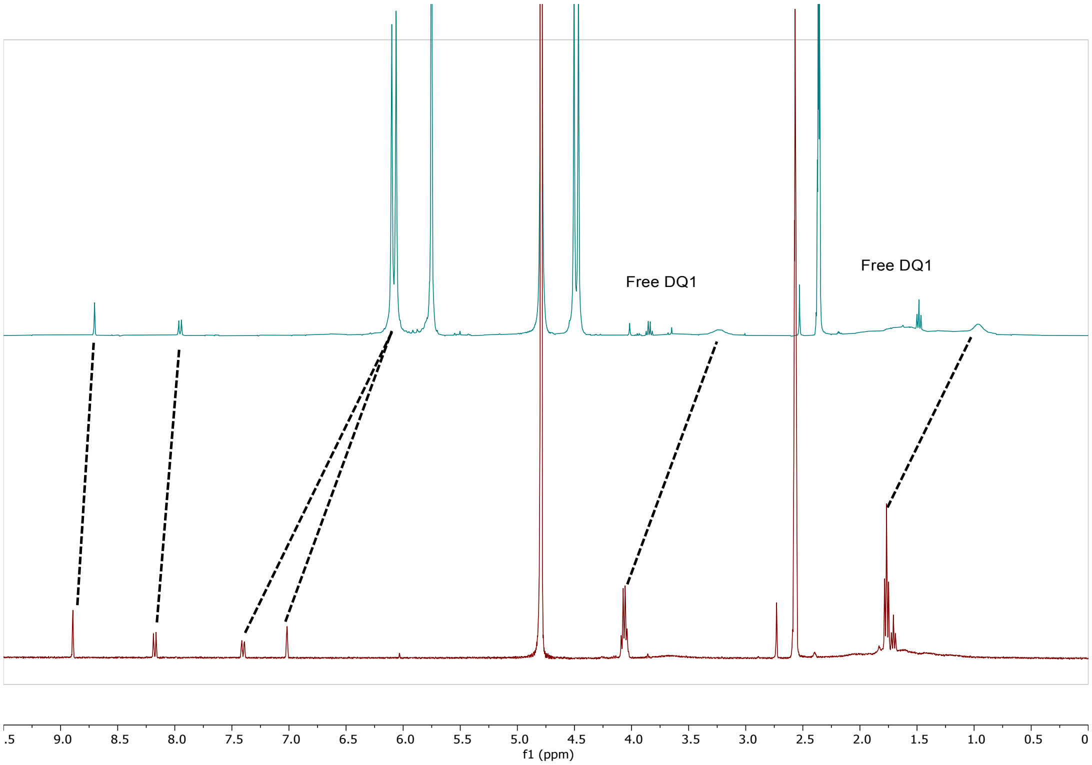

Figure S9. NMR-^{1}H spectra of **DQ1** (red) and **DQ1•CB7** (cyan) in a mixture acetonitrile-d3:deuterium oxide 1:1.

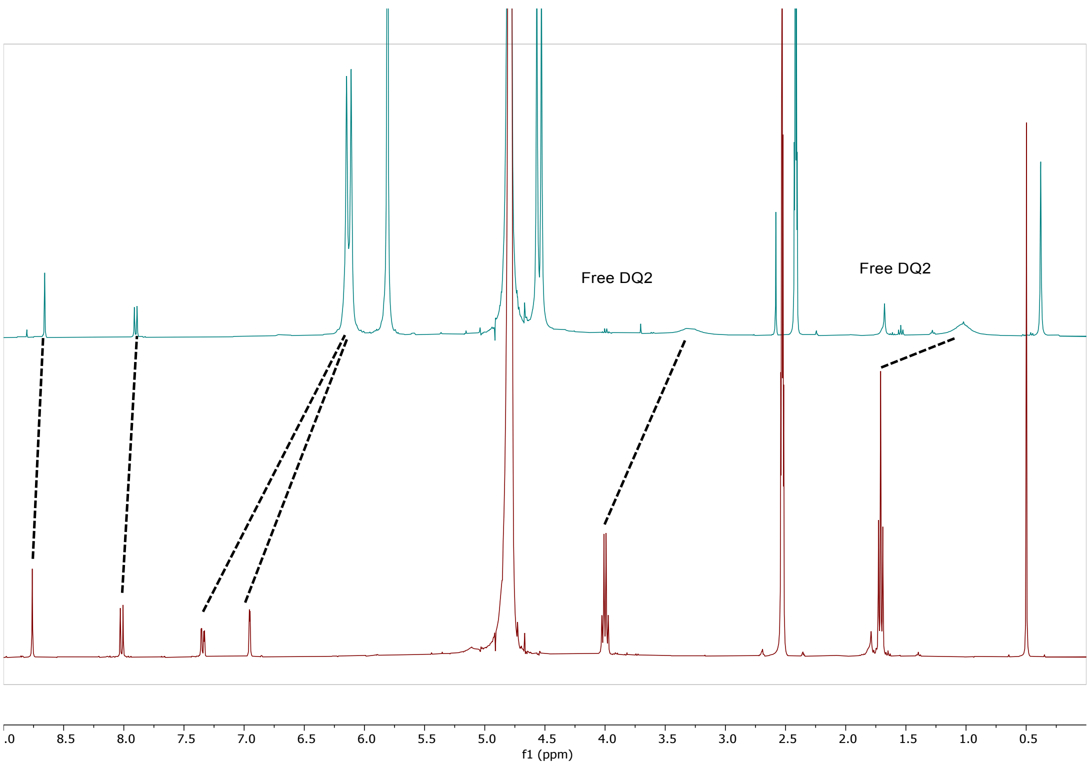

Figure S10. NMR-^{1}H spectrum of **DQ2** (red) and **DQ2•CB7** (cyan) in a mixture acetonitrile-d3:deuterium oxide 1:1.

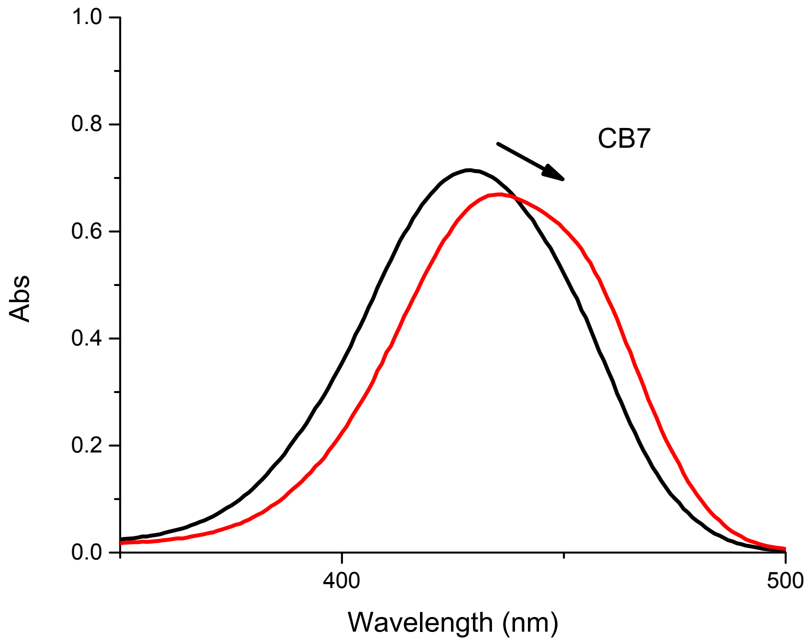

Figure S11. Variation of **DQ1** absorbance upon the addition of 10 equivalents of **CB7**.

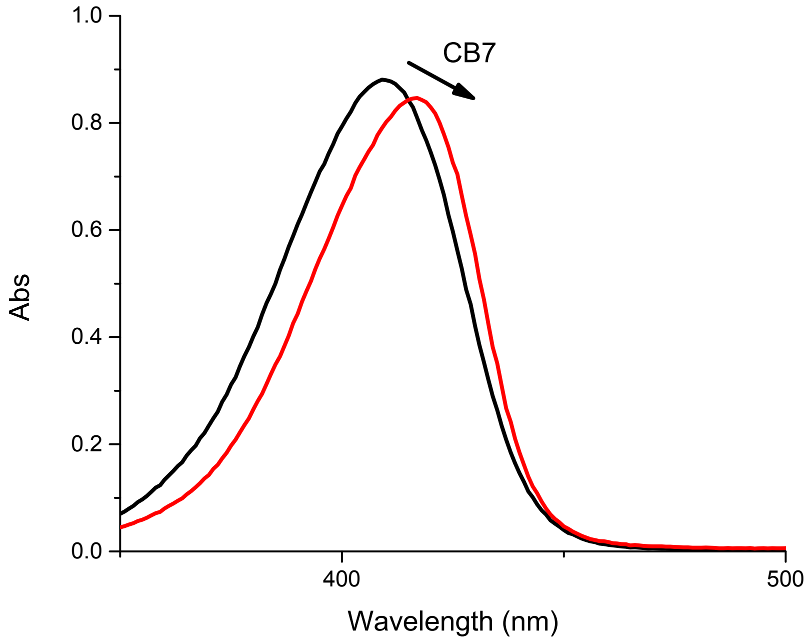

Figure S12. Variation of **DQ2** absorbance upon the addition of 10 equivalents of **CB**7.

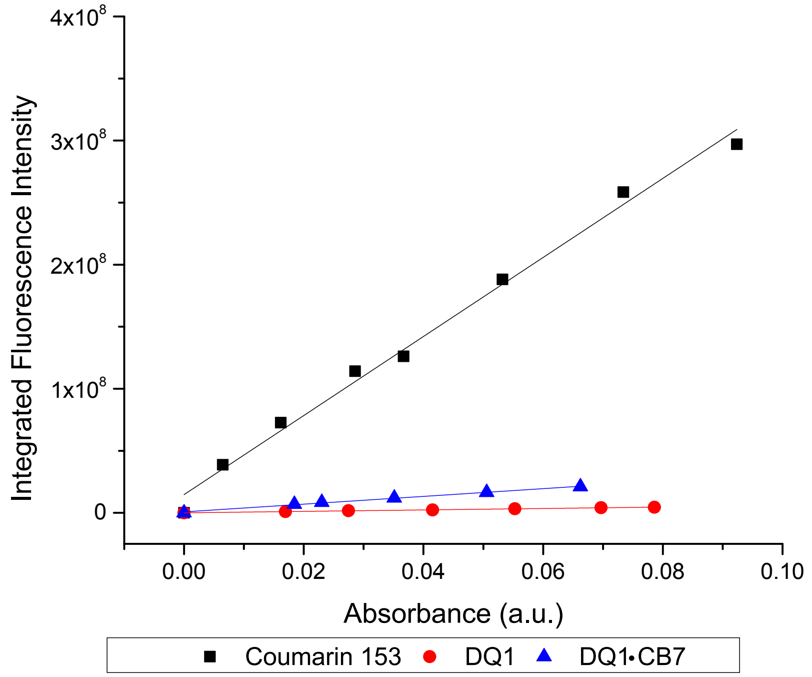

Figure S13. Dependence of integrated fluorescence intensity and absorbance for coumarin-153, **DQ1** and **DQ1•CB7**, used for the determination of quantum yield of the probe and complex. .

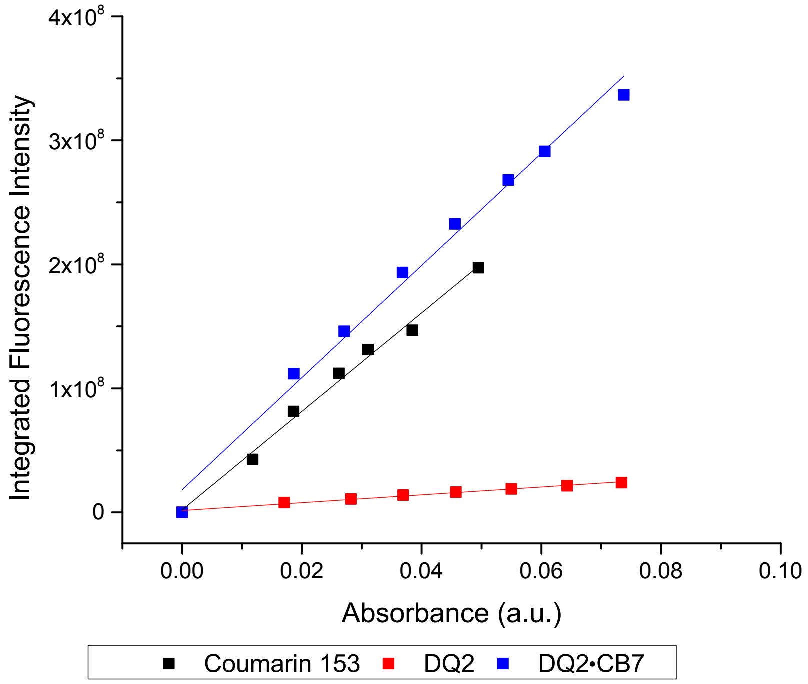

Figure S14. Dependence of integrated fluorescence intensity and absorbance for coumarin-153, **DQ2** and **DQ2•CB7**, used for the determination of quantum yield of the probe and complex. .

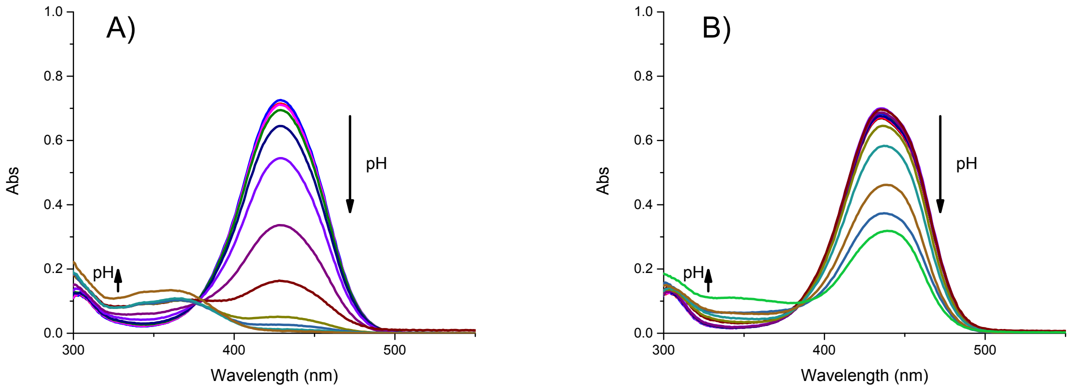

Figure S15. UV-Vis Spectra of A) **DQ1** and B) **DQ1•CB7** at different pH (0.05 – 5.86).

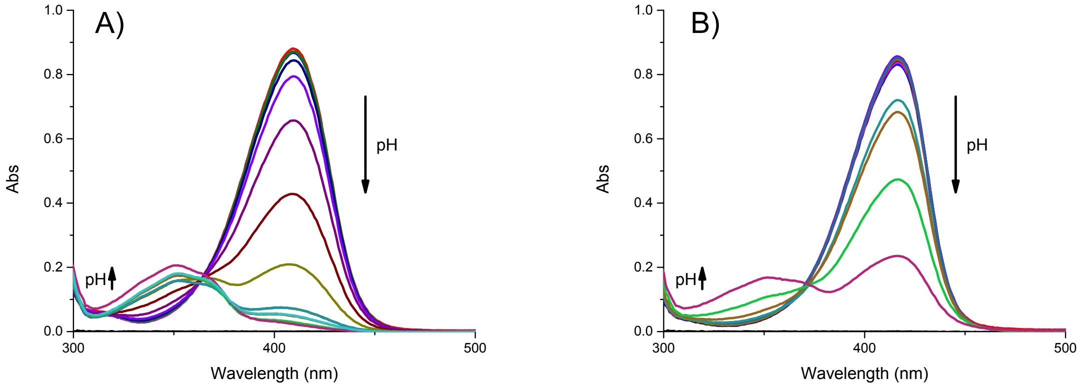

Figure S16. UV-Vis Spectra of A) **DQ2** and B) **DQ2•CB7** at different pH (0.05 – 5.86).

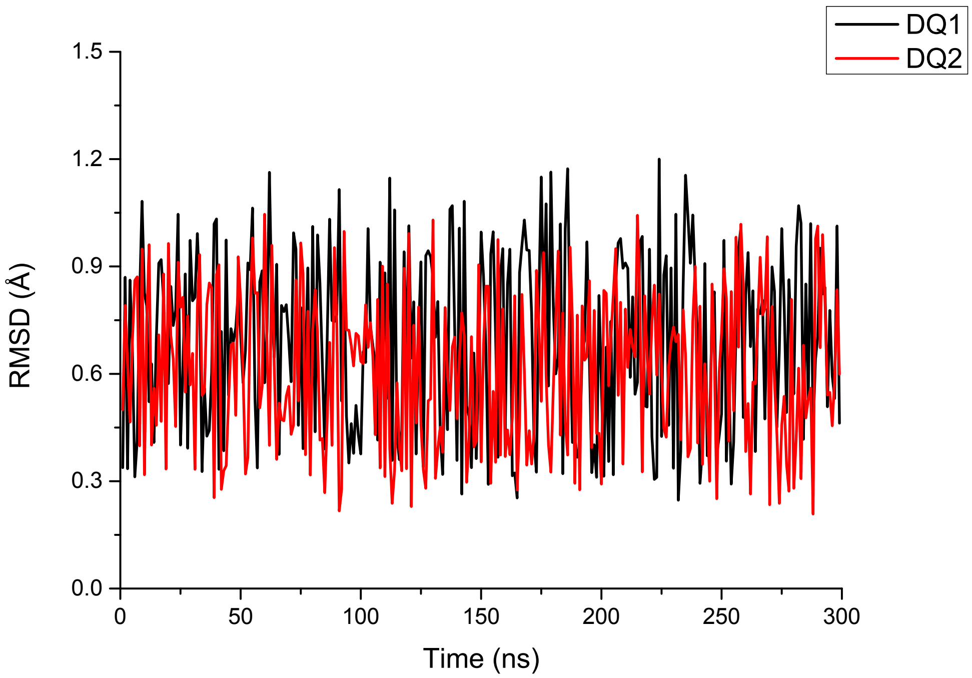

Figure S17. RMSD for **DQ1** and **DQ2** inside **CB7** during a 300 ns MD study.

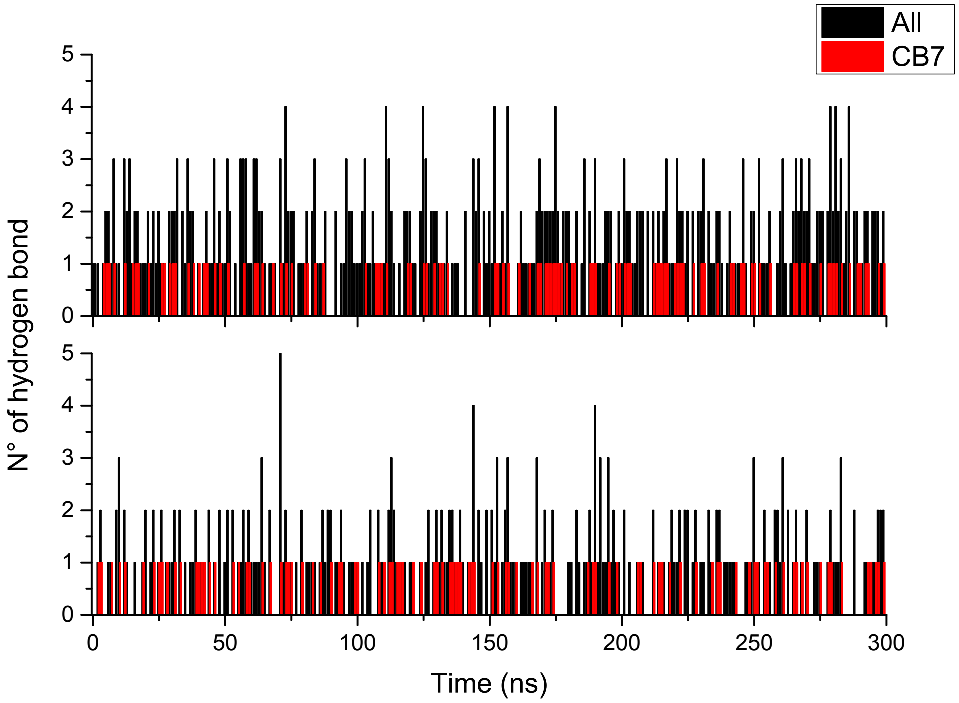

Figure S18. Hydrogen bonds formed between probe - **CB7** (red bars) and probe or **CB7** – solvent (black bars) A) **DQ1**, B) **DQ2** during a 300 ns MD study.

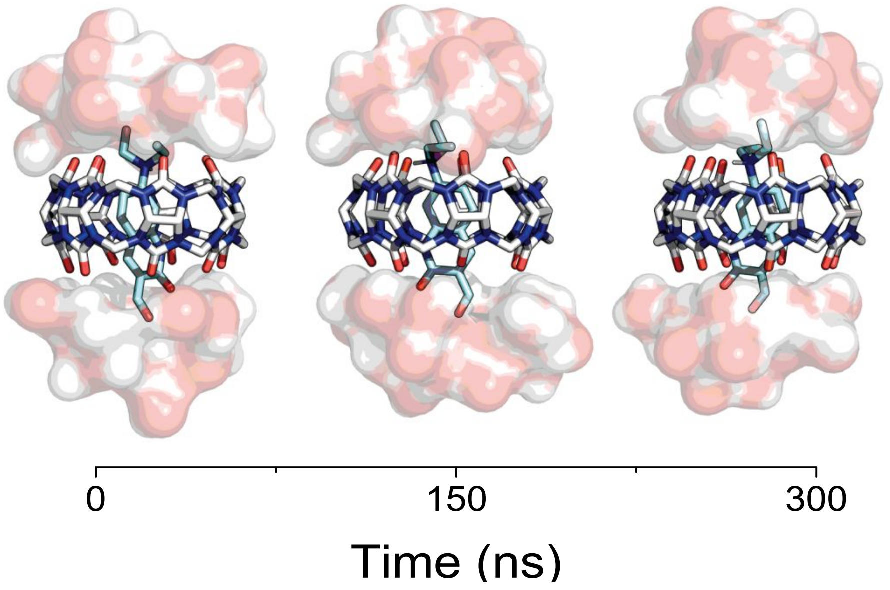

Figure S19. Conformations of **DQ1** inside **CB7** during a 300 ns MD study. The water molecules interacting with the probe and the macrocycle are shown as a surface.

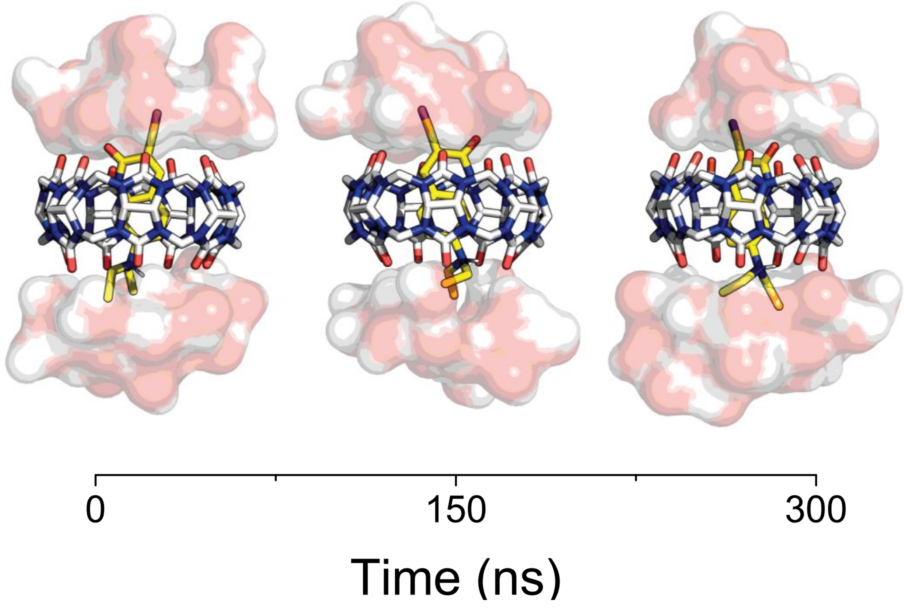

Figure S20. Conformations of **DQ1** inside **CB7** during a 300 ns MD study. The water molecules interacting with the probe and the macrocycle are shown as a surface.
